# Electrophysiological mechanisms underlying hypoxia‐induced deficits in visual spatial and non‐spatial discrimination

**DOI:** 10.14814/phy2.15036

**Published:** 2021-09-23

**Authors:** Qi Qiu, Pengpeng Lv, Yihao Zhongshen, Fengjuan Yuan, Xinjuan Zhang, Xiuzhu Zhou, Shanhua Li, Xiaonan Liu, Jiaxing Zhang

**Affiliations:** ^1^ Institute of Brain Diseases and Cognition School of Medicine Xiamen University Xiamen Fujian China; ^2^ Department of Clinical Medicine School of Medicine Xiamen University Xiamen Fujian China; ^3^ Department of Traditional Chinese Medicine School of Medicine Xiamen University Xiamen Fujian China; ^4^ Department of Gynecology and Obstetrics The First Affiliated Hospital of Xiamen University Xiamen Fujian China; ^5^ Institute of Psychology School of Public Policy Xiamen University Xiamen Fujian China

**Keywords:** EEG, ERP, high altitude, hypoxia, visuospatial discrimination

## Abstract

Impaired visual cognition in residents of hypoxic environment has been widely reported; however, the underlying electrophysiological mechanisms remain unclear. In this study, 23 college students underwent three sessions of a Clock task test before a 30‐day high‐altitude exposure (Test 1) and 1 week (Test 2) and 3 months (Test 3) after they returned to lowlands. The Clock task consists of a visual spatial angle and a visual non‐spatial color discrimination subtask. Simultaneously, electroencephalography (EEG) was recorded during the Clock task. The behavioral results showed that, compared with Test 1, accuracy in Test 2 was significantly decreased in both the Angle and Color tasks, and reaction time (RT) was significantly increased in the Angle task. The event‐related potentials results showed that, during both tasks amplitudes of the occipital N1 and P3 components during both tasks were significantly decreased in Test 2, compared with Test 1. Moreover, N1 amplitude was negatively correlated with RT and positively correlated with accuracy. Further time–frequency EEG analysis showed that theta power at occipital sites was significantly decreased in both tasks in Test 2, compared with Test 1, and was negatively correlated with RT in the Angle task. In Test 3, both the behavioral performance and EEG activity recovered to the baseline level in Test 1. These findings suggested that hypoxia impairs both visual spatial and visual non‐spatial discriminations, and these impairments can recover after subjects return to lowlands. Inhibition of brain electrophysiological activity in the visual cortex may explain the deficits in visual cognition.

## Graphical abstract

A first longitudinal electrophysiological study for high altitude (HA) visual cognitions. Both visual spatial and visual non‐spatial abilities impaired after HA exposure. Inhibition of event‐related potential N1 and P3 in visual cortex explains the visual impairment.

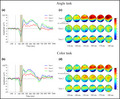

## 1 INTRODUCTION

Hypoxic challenge at high altitude (HA) usually puts individuals at risk for neurological impairments. A systematic literature review showed that visual cognitions are most easily impaired after people who live at sea level ascend to plateaus for anywhere from a day to dozens of days (Petrassi et al., 2012). For example, previous studies have shown that hypobaric hypoxia caused impairments in visuospatial memory (Cavaletti & Tredici, 1993; Hornbein et al., 1989; Kramer et al., 1993), visuospatial transformation (Denison et al., 1966), visual monitoring (Beer et al., 2017), visual orientation (Zhang, Ma, et al., 2018), visual search (Zhang, Zhang, et al., 2018), visual construction (Zhang et al., 2011), visuospatial executive (Sharma et al., 2014), visuospatial working memory (Ma et al., 2019; Ray et al., 2019; Yan et al., 2011), and color perception (Feigl et al., 2011; Karakucuk et al. 2004). However, the mechanisms underlying the impairments of visual cognitions remain unclear.

The effects of hypobaric hypoxia on brain neuronal activities were induced by auditory (Hayashi et al., 2005; Kida & Imai, 1993; Richardson et al., 2011; Thakur et al., 2011; Wesensten et al., 1993), somatosensory (Nakata et al., 2017), and attention (Ma et al., 2018, 2019; Wang et al., 2014; Zhang, Ma, et al., 2018; Zhang, Zhang, et al., 2018) tasks have been studied in subjects exposed to simulated altitude, rapid ascent to HA, or stayed at HA for several weeks to months. In these studies, event‐related potentials (ERPs), obtained by time‐locked averaging electroencephalography (EEG), were employed to evaluate electrophysiological processing of cognitive activity. Based on these data, we hypothesized that changed ERP components may be related to the hypoxia‐induced deficits in visuospatial cognition.

In most case, after a period of HA hypoxia exposure, people descend to lowlands and the altitude‐acclimatized brain will probably suffer from the stresses generated by reoxygenation and hyperbaric atmosphere, and trigger different pathophysiological signs, leading to transient or irreversible functional alterations. Previously, we have found changes in EEG powers and cortical gray matter in volunteers during hypoxia exposure at HA and the recovery of these changes after following reoxygenation at lowland (Fan et al., 2016; Zhao et al., 2016), which indicate certain behavioral and physiological changes induced by sojourn at altitude. Therefore, in this study, sea‐level college students, who volunteered for 30 days teaching experience on the Qinghai‐Tibet plateau, were recruited, and EEG recording during “Clock task” was conducted to investigate the meaning of electrophysiological changes. Moreover, time–frequency analysis of event‐related EEG data was also performed. Time–frequency analysis allows analyzing both the frequency of an event‐related oscillations and its evolution over time. The “Clock task” was employed to test visual and visuospatial abilities (Antonova et al., 2015; Sack et al., 2002). The Clock task consists of a visual spatial task (angle discrimination) and a visual non‐spatial task (color discrimination). The color discrimination task serves as a control task to visuospatial task. In Angle task, subjects need to evaluate the size of angles between the two clock hands; while in Color task, subjects just need to discriminate the color of the clock hands.

## 2 METHODS

### 2.1 Subjects

Twenty‐three healthy college students (12 females and 11 males, 19.4 ± 0.9 years, range 19–21 years) were recruited from Xiamen University. All subjects were lowland residents (<1500 m) and have been living at Xiamen (sea level, China) for at least 3 months. All the subjects had normal or corrected‐to‐normal vision. No subject was a smoker and was allowed access to alcohol. Subjects were excluded if they developed mountain sickness during their teaching period or had a documented neurological disorder and head injury. This study was approved by the Ethics Committee of the Xiamen University. Procedures were fully explained and all subjects provided written informed consent before participating in the study. Another 20 healthy college students of comparable age, sex, and education, were recruited from Xiamen University as controls for verification of the reliability of the EEG measurements and as controls in the behavioral test performed at three time points.

### 2.2 Plateau trip and experimental design

During the first 3 days, the subjects left Xiamen for Lhasa (3650 m), Tibet, China. After 1‐day stayed at Lhasa, the subjects spent 4 h traveling to Dangxiong city (4300 m), Tibet. During the entire period, all subjects stayed at Dangxiong as volunteer teachers. On the 29th day, they finished teaching work and descended to Lhasa. Three days later, they returned to Xiamen. At Dangxiong, the subjects had access to similar food and drink as that in Xiamen. All subjects had successfully finished teaching work, without the use of supplementary oxygen. A baseline set of physiological measurements and EEG recordings was initially acquired at Xiamen before ascent to Dangxiong (Test 1); the same set of tests was performed 1 week after subjects returned to Xiamen (Test 2); the final set of tests was performed after the subjects had been living at Xiamen again for 3 months (Test 3).

### 2.3 Physiological measurements

Physiological tests included the heart rate, blood pressure, hematological measure, arterial oxygen saturation (SaO_2_), and pulmonary function. Blood samples were taken in the morning between 07:00 and 07:30 a.m. De‐acclimation was tested after subjects returned to sea level at Test 2 and Test 3, respectively. Symptom score and diagnostic criteria for de‐acclimatization syndrome were adopted from He et al. (2013). De‐acclimatization symptom scores >14 indicates no de‐acclimatization symptom.

### 2.4 EEG recording

Thirteen subjects attended EEG study. Brain electrical activity was recorded via 128‐channel electrode cap using Ag/AgCl electrodes in AchView707‐Laptop software. The reference electrodes were placed in the bilateral auricular processes. EEG was recorded at a sampling rate of 500 Hz, applying <5 KΩ electrode impedances.

**FIGURE 1:**
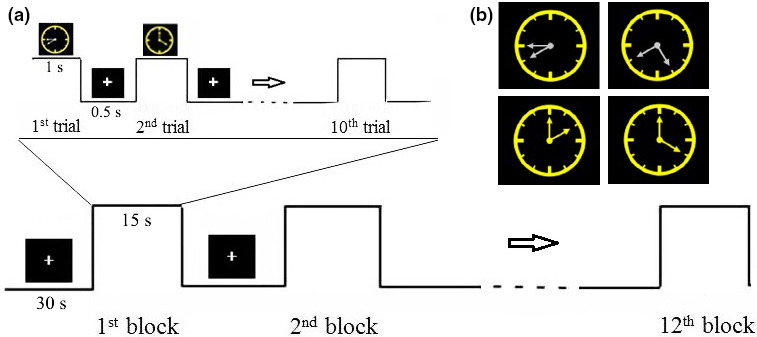
Stimuli and experimental procedure. (a) Example clocks with 30, 60, 90, and 120° between alternating white and yellow clock hands. (b) Paradigm for one block. Totally 12 blocks for the entire experiment

The Clock task, which has been designed previously (Antonova et al., 2015; Sack et al., 2002), was applied. The visual stimuli consisted of schematic analog clocks with a yellow face and two white or yellow hands presented on a black computer screen. The angle between the clock hands varied in steps of 30°. Subjects needed to discriminate angle and color. In the angle discrimination task, clock with small angle of 30° or 60° is the “target” and clock with large angle of 90°, 120°, or 150° is the “non‐target.” In the color discrimination, color with white hand is the “target” and clock with yellow hand is the “non‐target.” Stimuli were programmed for display with E‐prime software (version 3.0, Psychology Software Tools) and presented on an LED monitor. Subjects were sitting comfortably in a darkened, sound‐proofing, and electrically shielded house, and a chin rest was used to avoid head movement. They were instructed to press the button in the presence of target (key 1) and non‐target (key 2). Although the assignment of target and non‐target to the stimulus categories was random, subjects were explicitly instructed to consider the deviant and small angle as target in the Angle task and white hand as target in the Color task. The task consisted of 12 blocks, with 10 trials in each block (Figure 1). In each trial, five angles and five colors were present. Between blocks and trials, a white “+” were present. Before the formal test, subjects were provided for sufficient practice to make sure that the accuracy rate of task reached more than 80%. During the EEG recording, the reaction time (RT) and accuracy of performing Clock tasks were recorded at the same time.

### 2.5 EEG data preprocessing

EEGLAB/ ERPLAB toolbox under the MATLAB (The MathWorks, Inc.) was used for data analysis. The row data were calculated to the common average reference and filtered with 0.1–30 Hz. To define the optimal end of time window for the analysis of EEG epochs, the distribution of RT from each subject was inspected. As a result, a time window of −200 to 1000 ms from stimulus onset was confirmed. Eye movement artifact was corrected by moving the vertical and horizontal eye movements identified by an independent comment analysis. In addition, epochs containing further artifacts were discarded in a semiautomatic artifact inspection (maximum allowed voltage step per sampling of 100 μV, a maximum difference in values in intervals of 200 ms of 500 μV, and a maximum and minimum amplitude allowed of −200 μV to 200 μV). Finally, only blocks with correct responses were averaged and segmented into stimulus‐locked ERP negative potential (N1) and positive potential (P3) components. The mean voltage of N1 component over 130–180 ms at the O1, OZ, and O2 electrodes and P3 component over the 300–500 ms at the P1, PZ, and P2 electrodes was measured to observe the early and late visuospatial processing in the Color task and Angle task, respectively.

### 2.6 Time–frequency EEG analysis

The time–frequency representations (TFRs) of power were computed for EEG segments time locked from 200 ms pre‐stimulus to 1000 ms post‐stimulus onset with frequency ranging from 1 to 30 Hz in steps of 1 Hz and a sliding time window of 900 ms in steps of 20 ms. The data in each time window were multiplied with a Hanning taper, followed by a Fourier transform. The TFRs were averaged over trials for each condition per subject, and then normalized to mean power over baseline interval (−200 to 0 ms) using a decibel (dB) transform. These analyses were performed using the FieldTrip toolbox under the MATLAB.

### 2.7 Data analyses

All data were analyzed with SPSS 25.0. Paired samples *t*‐test was performed on the physiological measurements, performances of Clock task, and ERP recording. Pearson correlations were used to assess the correlations of RT and accuracy with time–frequency power, N1 amplitude, and P3 amplitude. Statistical significance was set at *p* < 0.05.

## 3 RESULTS

### 3.1 Physiological characteristics

Red blood cells (5.1 ± 0.8 vs. 4.9 ± 0.5, *p* = 0.005) and hemoglobin (42.7 ± 4.3 vs. 40.2 ± 3.1, *p* = 0.008) were significantly increased in Test 2 compared with Test 1. No significant differences were found in the hematological measurement in Test 3 compared with Test 1. No significant changes in vision were detected in both Test 2 and Test 3 compared with Test 1. De‐acclimatization symptom scores were <14 in all subjects.

### 3.2 Behavioral performances

There was a significant decreased accuracy in both Angle task and Color task and a significant increased RT in Angle task in Test 2 compared with Test 1 (Table 1). No significant differences in RT and accuracy were found in Test 3 compared with Test 1.

**TABLE 1:** Behavioral results in Test 1, Test 2, and Test 3

	Test 1	Test 2	Test 3	P1	P2	t 1	t 2
RT (ms)							
Angle task	555.77 (50.54)	642.41 (94.42)	582.13 (76.58)	0.027	0.408	2.364	0.869
Color task	520.29 (62.96)	560.25 (76.20)	520.25 (72.02)	0.113	0.999	1.653	0.01
Accuracy (%)							
Angle task	91% (0.03)	86% (0.04)	91% (0.03)	0.014	0.825	3.093	0.267
Color task	95% (0.02)	89% (0.07)	94% (0.01)	0.028	0.081	2.630	1.830

Note

t 1 and P1: Test 2 versus Test 1; t 2 and P2: Test 3 versus Test 1. Data are shown as mean (SD).

Abbreviation: RT, reaction time.

### 3.3 N1 and P3 powers

Thirteen subjects attended EEG study. Three subjects were excluded because of their frequency eye movement, excessive artifacts in the electroencephalogram, or data lost. Eventually, only EEG data of 10 subjects were analyzed.

#### 3.3.1 N1 and P3 amplitude

**FIGURE 2:**
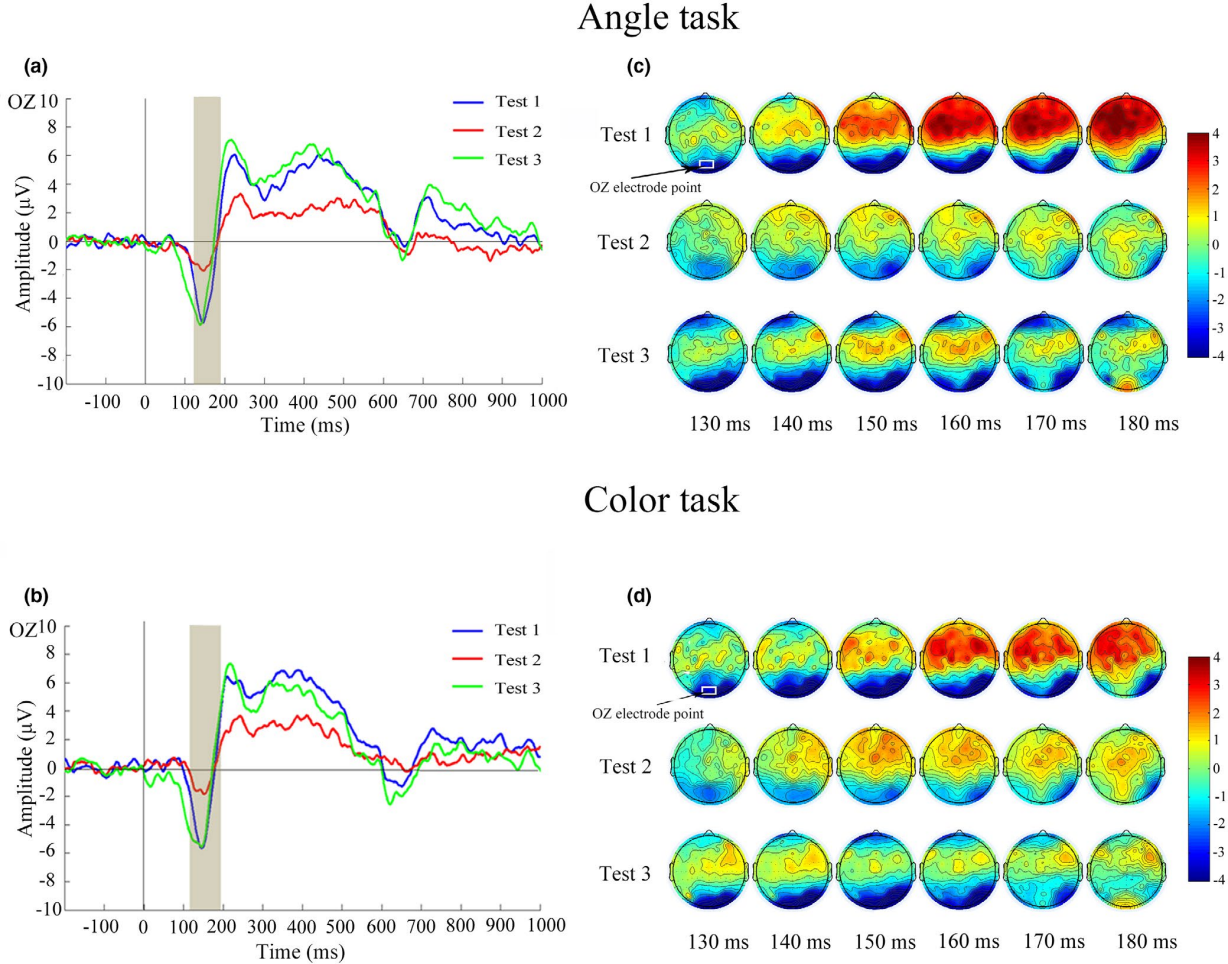
Waveforms of N1 (130–180 ms) and corresponding scalp topographies (generated every 10 ms from 130 to 180 ms) in the three tests. (a) Averaged ERPs at OZ electrode in the three tests elicited by Angle task. (b) Averaged ERPs at OZ electrode in the three tests elicited by Color task. (c) Scalp topographies within the N1 time window in the three tests under the Angle task. (d) Scalp topographies within the N1 time window in the three tests under the Color task. ERP, event‐related potential

**FIGURE 3:**
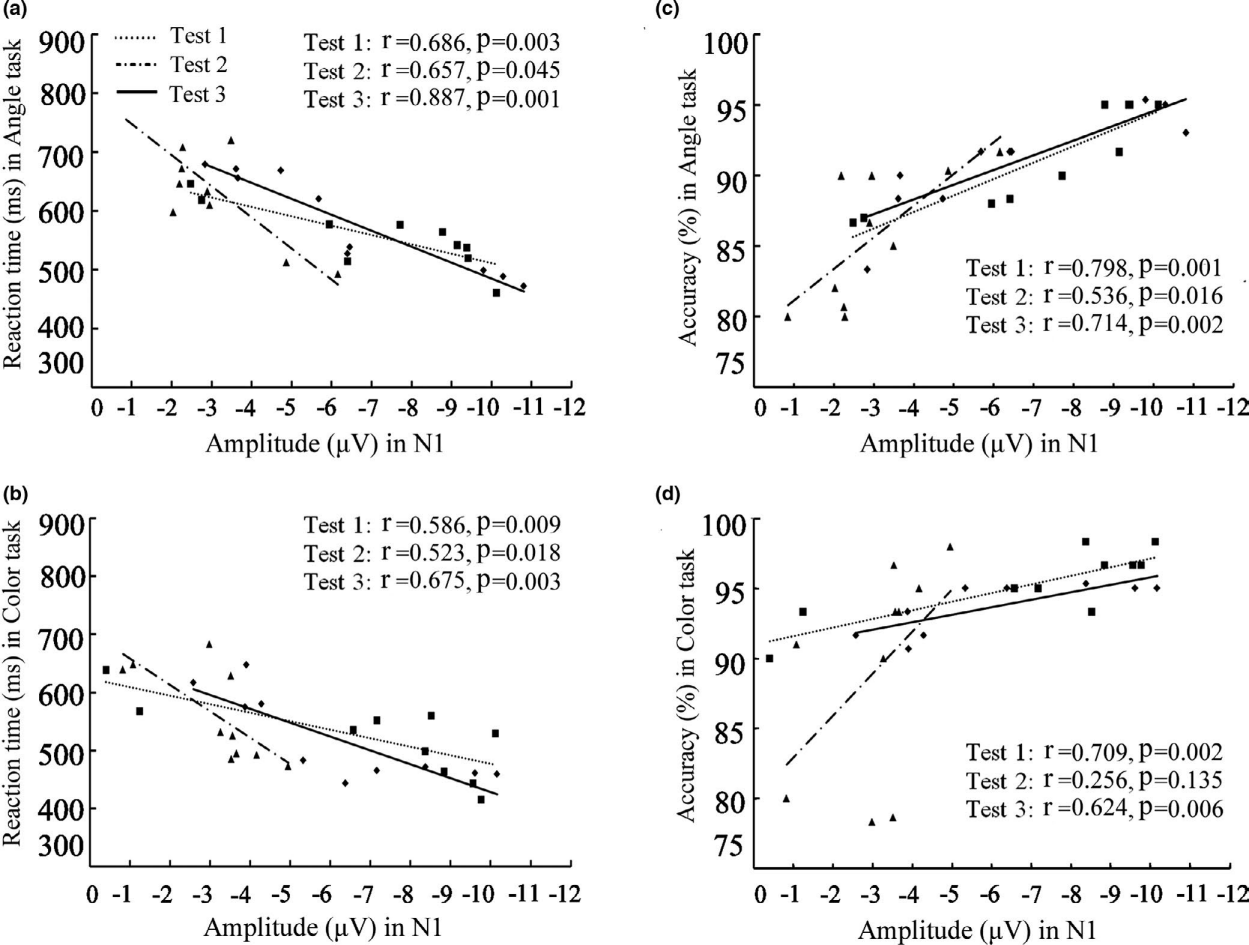
Correlation of N1 amplitude value with RT (a, b) and accuracy (c, d) in the three tests. RT, reaction time

**FIGURE 4:**
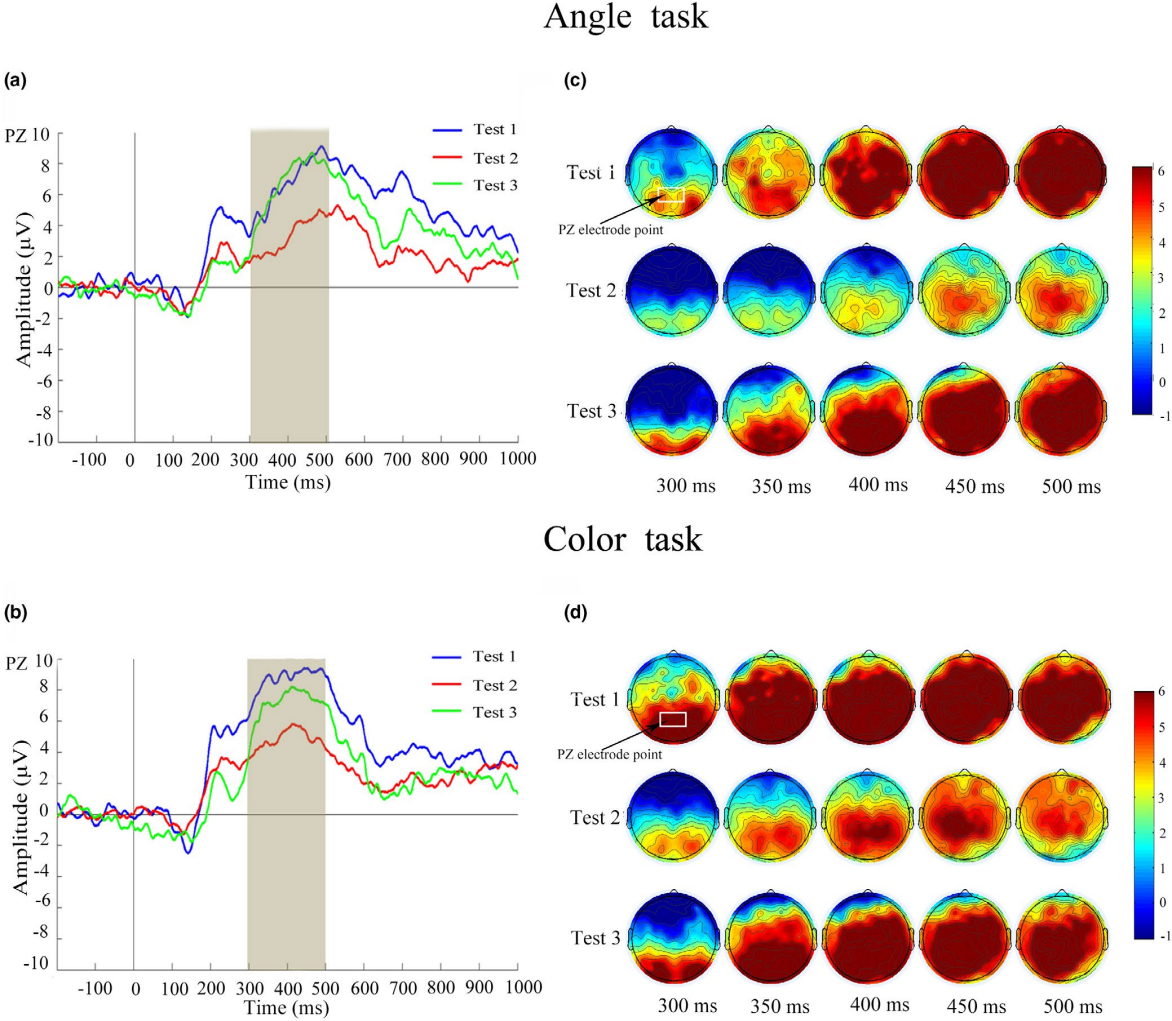
Waveforms of P3 (300–500 ms) and corresponding scalp topographies (generated every 50 ms from 300 to 500 ms) in the three tests. (a) Averaged ERPs at PZ electrodes in the three tests under the Angle task. (b) Averaged ERPs at PZ electrodes in the three tests under the Color task. (c) Scalp topographies within the P3 time window in the three tests under the Angle task. (d) Scalp topographies within the P3 time window in the three tests under the Color task. ERPs, event‐related potentials

**FIGURE 5:**
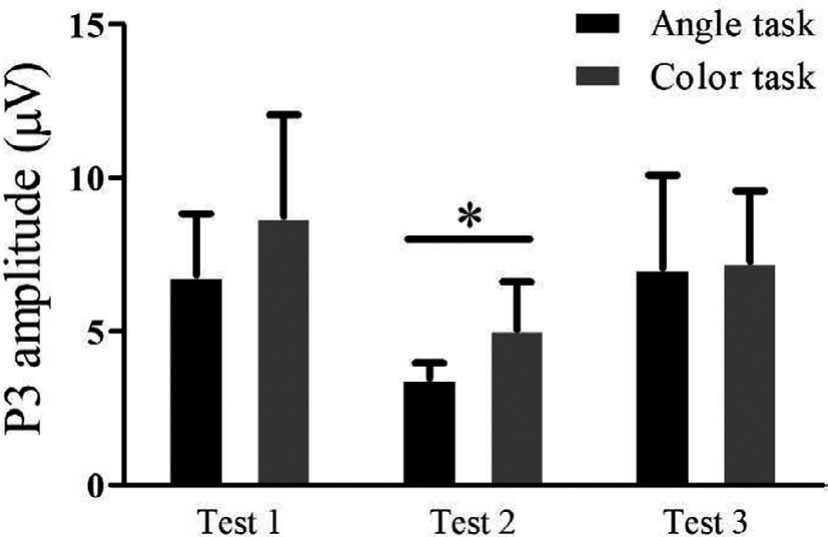
P3 amplitude elicited by the Angle task and Color task. In Test 2, P3 amplitude was significantly lower in Angle task than in Color task. *p < 0.05

N1 amplitude values at occipital OZ, O1, and O2 electrodes and P3 amplitude values at parietal PZ, P1, and P2 electrodes during Angle task and Color task are shown in Table 2. In Test 2 compared with Test 1, N1 and P3 amplitude values at three electrodes were significantly decreased; In Test 3 compared with Test 1, N1 and P3 amplitude values at three electrodes showed no significant differences. Figure 2 shows waveform and scalp topography of averaged N1 at OZ electrode in the three tests in Angle task and Color task. N1 amplitude was negatively correlated with RT in all three time point tests in both Angle task and Color task; N1 amplitude was positively correlated with accuracy in Angle task in all three time point tests, and positively correlated with accuracy in Color task in Test 1 and Test 3 (Figure 3). Figure 4 shows waveform and scalp topography of averaged P3 at OZ electrode in the three tests in Angle task and Color task. Moreover, in Test 2, there was significantly lower P3 amplitude in Angle task compared with Color task (Figure 5).

**TABLE 2:** N1 amplitude values at occipital OZ, O1, and O2 electrodes and P3 amplitude values at parietal PZ, P1, and P2 electrodes during Angle task and Color task

	Test 1	Test 2	Test 3	P1	P2	t 1	t 2
N1 amplitude (μV)							
Angle task							
OZ electrode	−6.59 (2.32)	−2.93 (1.83)	−6.12 (2.31)	0.001	0.644	5.534	0.644
O1 electrode	−6.65 (2.80)	−2.95 (1.46)	−5.54 (2.79)	0.003	0.249	4.032	1.231
O2 electrode	−9.60 (3.59)	−3.44 (1.74)	−7.82 (3.15)	0.001	0.647	5.931	1.505
Color task							
OZ electrode	−6.32 (2.92)	−2.93 (1.46)	−6.09 (2.30)	0.001	0.840	5.468	0.208
O1 electrode	−5.93 (3.79)	−2.91 (1.59)	−5.40 (2.31)	0.029	0.640	2.591	0.484
O2 electrode	−9.29 (4.00)	−3.34 (1.04)	−7.34 (3.13)	0.001	0.240	0.354	1.258
P3 amplitude (μV)							
Angle task							
PZ electrode	10.19 (2.31)	5.86 (1.56)	9.84 (3.32)	0.001	0.794	4.969	0.268
P1 electrode	10.20 (2.02)	6.01 (1.10)	10.28 (3.52)	0.001	0.961	5.769	0.050
P2 electrode	10.26 (2.30)	5.63 (1.40)	10.41 (3.43)	0.001	0.918	5.964	0.105
Color task							
PZ electrode	4.91 (2.21)	2.68 (1.28)	3.37 (3.39)	0.009	0.276	3.312	1.159
P1 electrode	4.88 (2.02)	2.59 (1.10)	3.68 (3.52)	0.017	0.405	2.917	0.873
P2 electrode	5.98 (1.96)	2.61 (2.04)	4.15 (3.41)	0.001	0.252	4.930	1.225

Note

P1: Test 2 versus Test 1; P2: Test 3 versus Test 1; t 1: Test 2 versus Test 1; t 2: Test 3 versus Test 1. Data are shown as mean (SD).

#### 3.3.2 N1 and P3 latency

No significant differences were found in both Angle task and Color task in Test 2 compared with Test 1 and in Test 3 compared with Test 1. There were no significant differences between Angle task and Color task in N1 or P3 latencies at all three time point tests.

### 3.4 Theta power

**FIGURE 6:**
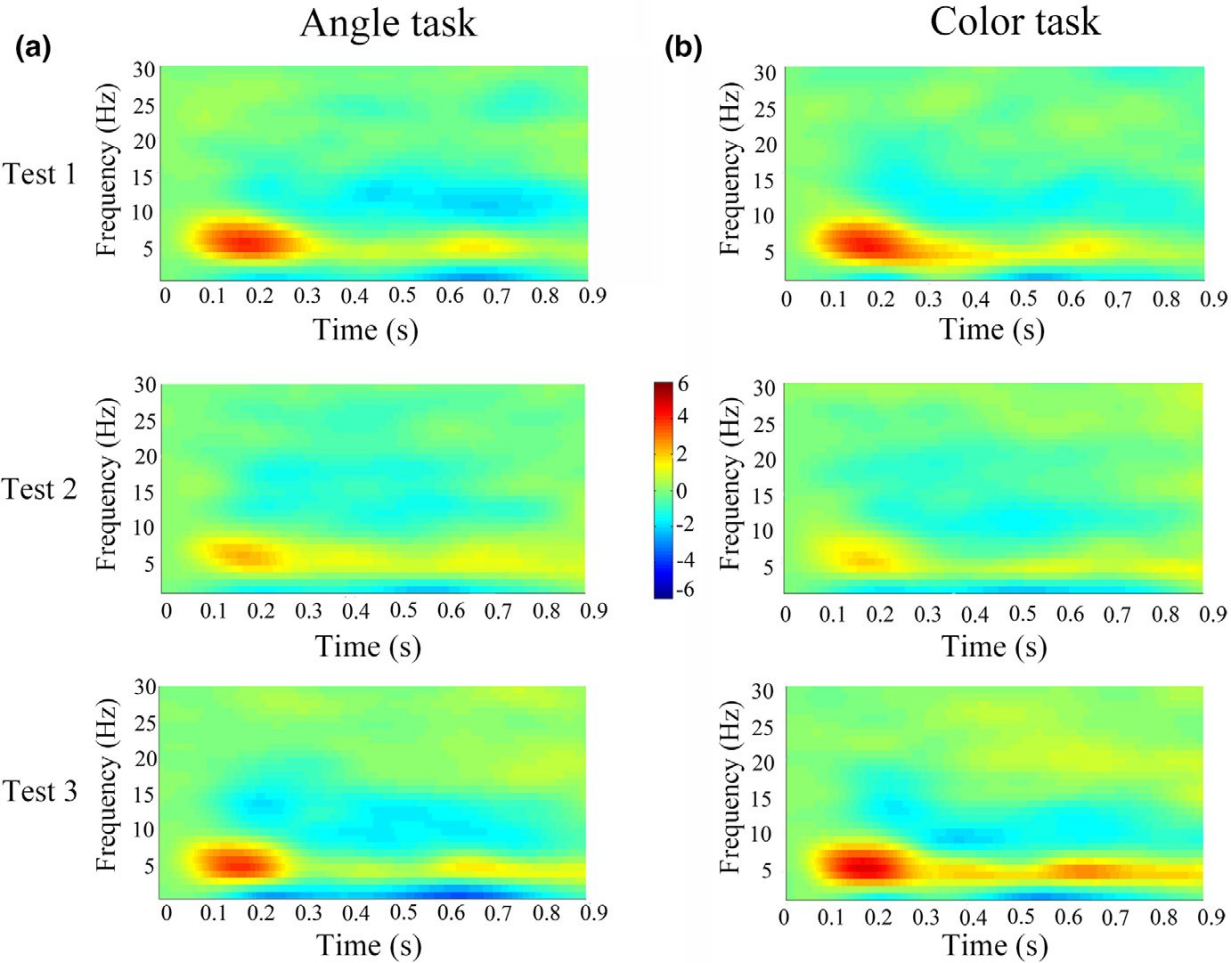
The time–frequency representations of EEG power locked from 200 ms pre‐stimulus to 1000 ms post‐stimulus onset, at frequency ranging from 1 to 30 Hz in steps of 1 Hz. The power of the theta band (4–8 Hz) in the 130–180 ms time window at the occipital lobe was significantly lower in both Angle task (a) and Color task (b). EEG, electroencephalography

In Test 2 compared with Test 1, the theta power (4–8 Hz) in the 130–180 ms time window at the occipital lobe was significantly decreased in both Angle task (*p* = 0.024) and Color task (*p* = 0.014) (Figure 6). No significant changes were measured in alpha, beta, or delta powers. In Test 3 versus Test 1, no significant changes were measured in alpha, beta, theta, or delta powers in both Angle task and Color task.

**FIGURE 7:**
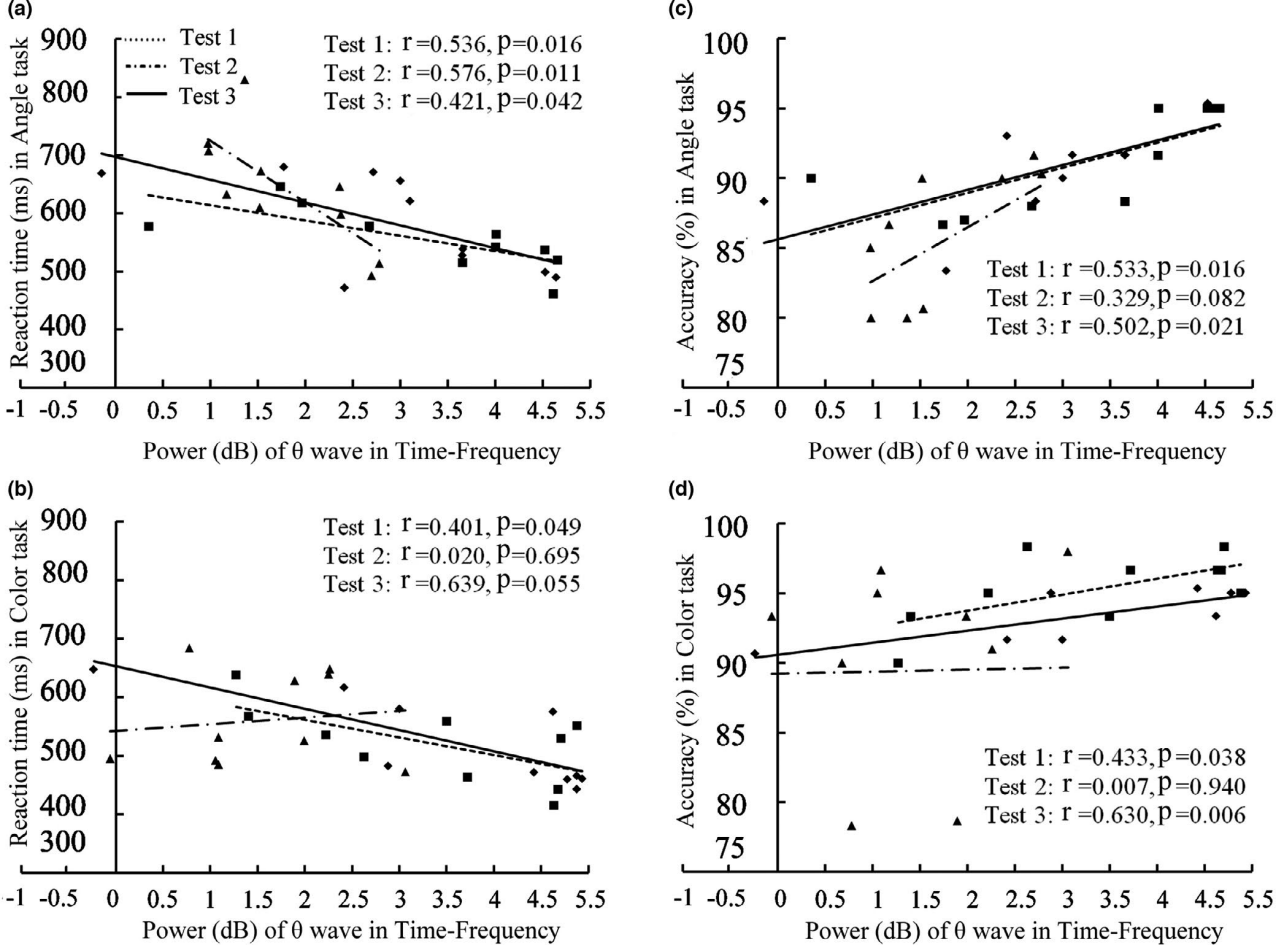
Correlation of power of theta wave in time–frequency with RT (a, b) and accuracy (c, d) in the three tests. RT, reaction time

The power of theta frequency was negatively correlated with RT and positively correlated with accuracy in Test 1 and Test 3 (Figure 7). In Test 2, the negative correlation was existed between power of theta frequency and RT in Angle task (Figure 7a).

## 4 DISCUSSION

This study showed a significantly decreased accuracy and a significantly increased RT in the performance of Angle task immediately after subjects descended from HA. At this time point, we also tested a significantly decreased accuracy in Color task, and meanwhile, impaired amplitude but not latency of the N1 and P3 component of the ERP was elicited by Angle task and Color task. N1 amplitude showed a negative correlation with RT and a positive correlation with accuracy in both Angle task and Color task. P3 amplitude was significantly lower in Angle task compared with Color task. Moreover, after 30‐day HA exposure, the theta power in the 130–180 ms time window was significantly lower in the Angle task and Color task and theta power was negatively correlated with RT in Angle task. After subjects returned to sea level for 3 months, the behavioral performances and ERP measurements recovered to basal level. The reliability of the cerebral measurements that could be affected by EEG instrument‐related factors such as recording–re‐recording using the same procedure was verified and the learning effect in the behavioral test was negated, as in sea‐level controls, no differences in EEG and behavioral measurements were found between three test time points.

In our study, 1 week after subjects returned to sea level, time–frequency EEG analysis showed only theta power at occipital sites was significantly decreased during performances of both Angle task and Color task. In our previous study, we recorded EEG in soldiers during eyes‐closed resting conditions, and found that after 1‐month HA (3800 m altitude) exposure and descending to lowlands 1 week, in the posterior parietal cortex, right posterior temporal cortex, and occipital cortex, alpha power decreased, beta power remained increased, while delta and theta power recovered to the baseline level (Zhao et al., 2016). In this study, the behavioral performances and ERP measurements recovered to basal level 3 months after subjects returned to sea level, which was consistent with our another study, in which cortical thickness in visual cortex increased in subjects who had a 30‐day teaching in Qinghai–Tibet plateau but cortical measurements recovered to basal level 2 months after HA exposure (Fan et al., 2016). The conclusion can be drawn from these three studies is that the different EEG powers were affected by hypoxia/reoxygenation, depending on subjects were under resting state or working state, and brain functional change has its structural basis.

In this study, decreased accuracy in the performance of Angle task and Color task was detected after HA exposure for 1 month, suggesting that HA hypoxia impaired both visual spatial and non‐spatial tasks. Angle task needs subjects to evaluate the size of angles between two clock hands, while Color task just needs subjects to discriminate the color of the clock hands while ignoring angle size. From Table 2, we can find that subjects spent more time, with lower accuracy, in conducting Angle task compared with Color task, and accordingly, N1 and P3 amplitudes in Angle task were higher than Color task. These data suggest that it takes more effort for subjects to perform Angle task than Color task. After a 30‐day hypoxia, significantly increased RT was found in the performance of Angle tasks but not in Color task and P3 amplitude was significantly lower in Angle task than Color task, which suggests spatial information processing may suffer more from hypoxia than from non‐spatial information processing.

In this study, at the early processing stage, N1 amplitude was significantly decreased at the occipital cortices after a 30‐day HA exposure compared with before ascent to HA. Moreover, N1 amplitude was negatively correlated with RT and positively correlated with accuracy in both Angle task and Color task across all three time point tests. Therefore, the decrease in N1 amplitude may underlie the mechanism of deficits in visuospatial and non‐visuospatial behavior. N1 has been shown to reflect the visual processing of spatial information (Heinze et al., 1994; Wascher et al., 2009) and is enhanced by visuospatial tasks (Di‐Russo et al., 2010; Hillyard & Anllo‐Vento, 1998; Lange et al., 1999; Vogel & Luck, 2010). The relationship between N1 amplitude and RT of behavior has been presented by Bahramali et al. (1998). The N1 component, which reflects the operation of discrimination processing, had highly reliable and focused activity in the occipital cortex (Jens‐Max et al., 2002). The decreased N1 amplitude in visual stimuli task suggests the less sensory gain of visual attention. The decreased occipital N1 component was also found in Parkinson's disease when patients were doing visual oddball test (Wang et al., 2001), in patients with traumatic brain injury in response to alerting visuospatial cues (Hill‐Jarrett et al., 2015), and in older population following the visuospatial stimulus (Curran et al., 2001). In consistent with our findings in visual ERP, the increased N1 latency of visual evoked potential (VEP) was observed in volunteers during the 3rd weeks of their stay at HA of 3500 m (Thakur et al., 2005) and in mountain climbers (Singh et al., 2004; Wohns et al., 1987). VEP amplitude was also reduced in subjects after 9–12 days of hypoxic exposure (Forster et al., 1975).

P3 occurs after presentation of a stimulus, requiring detection, counting, or cognitive processing (Picton, 1992). In our study, the late processing stage of P3 amplitude in the Angle and Color tasks at visual cortex was reduced after a 30‐day HA exposure, which indicates less remain of an effective index of mental resource and the degree of brain activity for current information processing was lower. Patients with traumatic brain injury exhibited reduced P3 amplitude to alerting visuospatial cues (Hill‐Jarrett et al., 2015). Change in P3 scalp topography induced by performing a visual classification was recorded in patients with transient global ischemia (Mecklinger et al., 1998). Decreased P3 amplitude to visual search was found in HA immigrants who had lived in HA of 3650 m for more than 3 years (Ma et al., 2018) and decreased P3 amplitude to auditory stimulus has been recorded by following a rapid ascent to simulated 4300 m altitude (Wesensten et al., 1993).

Theta frequency is associated with intake of sensory information, memory, synaptic plasticity, and top‐down control long‐range synchronization (Uhlhaas & Singer, 2013). In our study, the theta power was negatively correlated with RT and positively correlated with the accuracy both in the Angle task and Color task under normal behavioral performance before ascending and 3 months after subjects returned to lowlands, which indicates a high relationship of theta frequency with visual tasks and supports a role for theta frequency in visual attention (Spyropoulos et al., 2018). Furthermore, we found that the decreased theta power in the occipital area during Angle task and Color task after a 30‐day HA exposure, and theta power was negatively correlated with RT in Angle task. Therefore, the decreased theta power could be responsible for the impaired performance, especially in the Angle task. A number of studies have shown an association between theta frequency and cognition. Theta frequency showed a greater activation in the occipital region (Cartier et al., 2012) and decreased in patients with Parkinson's disease during the visuospatial task (Eichelberger et al., 2017). Reduces of theta oscillatory activity on cognitive load are common among schizophrenia, Alzheimer's disease, attention deficit hyperactivity disorder, Parkinson's disease (Güntekin & Başar, 2014), and older adults (Lithfous et al., 2015).

Visual ability is always directly affected by hypoxia. Hypoxic injury to the eye has been reported to play a significant role in visual impairment. The retina consumes oxygen more rapidly than other tissues and is sensitive to hypoxia (Country, 2017). Eye diseases such as retinal vascular lesions, cataracts, and blindness are often seen in Tibetans on the Qing‐Tibet plateau (Morris et al., 2006; Wang et al., 2013; Wang & Bai, 2011), and eye disturbance can affect the transmission of visual information to the cerebral cortex. Cones might be more sensitive to reduced oxygen compared to rods (Kurihara et al., 2016). It has been shown that chronic hypoxia can induce pathological vessel growth and cone degeneration (Barben et al., 2018). Therefore, damaged function of retinal photoreceptor cells may be involved in the deficits of visual ability.

Hypoxia can directly act on nerve cells in visual cortex. A reduced neuronal activity (Rostrup et al., 2005) and an increase in cerebral metabolic rate of oxygen occurred in the visual cortex during exposure to acute hypoxia (Vestergaard et al., 2016). Changed resting‐state neuronal activity in visual cortex has been found in lowland soldiers, who have garrisoned the frontiers in Qinghai–Tibet Plateau for 2 years (Zhang et al., 2017) and for 1 month (Zhao et al., 2016). Changed structures in occipital visual cortices may also contribute to ERP changes. An increase in cortical thickness in visual cortex was found in sea‐level college students, who had a 30‐day teaching in Qinghai–Tibet plateau (Fan et al., 2016). Increased fiber connectivity between the bilateral visual cortices was found in subjects after HA exposure for 2 years (Chen et al., 2016), which was explained as a central compensation. Compared with Han population living at sea level, Han immigrant descendants (Zhang et al., 2010) and Tibetan natives (Wei et al., 2017) in Qinghai–Tibetan Plateau showed impairments in the occipital visual cortices. Moreover, ultraviolet light has been shown to alter neuronal activity in the cortical structures involved in visual processing (Amir & Robinson, 1996).

In adult brain, the regional changes in cerebral perfusion may contribute to both functional and structural alterations. The regional cerebral perfusion was correlated with the activity of the cortical neurons in the region (Sokoloff, 1977). At HA, total CBF in the internal carotid and vertebral flow increases steadily with altitude, and it soon recovers to usual level with time (Ainslie & Ogoh, 2010). However, after subjects return to lowlands, CBF may decrease. For example, Joynt et al. (2008) found that the carotid blood flow was significantly lower than normal baseline after 4 h of reoxygenation for 2 h of following hypoxia (10%–15% O_2_); In rats, CBF showed modest increases during 60 min of hypoxia and returned to baseline during reoxygenation (Fujisawa et al., 1999). In addition, when descending to lowlands, HA residents often present HA deadaptation, which may affect neurophysiologic activity. In fact, mental symptom such as dizziness, hypersomnia, malaise, and hypophrenia are often seen in the subjects with HA deadaptation (Zhou et al., 2012).

Impaired attention during hypoxic exposure may also contribute to visual cognitive deficits. The effects of HA on attention capacity have been largely described. Slowing of the signal processing was detected in sea‐level subjects after 1 and 6 months of mountain climber stayed in the Eastern Himalayas (Thakur et al., 2011). Psychomotor vigilance reaction speed was slower during HA exposure (Pun et al., 2018). Visual search ability was reduced after 8 h of hypoxia (Stivalet et al., 2000). The parietal distributed P3, which is involved in maintaining attention, was the ERP component most significantly affected by hypoxia (Hayashi et al., 2005; Wesensten et al., 1993). The smaller P3 amplitude has been found in the population exposed to HA for 3 years when they performed spatial attention discrimination task (Wang et al., 2014).

There are several limitations in this study. One limitation was that the number of subjects was small. Although the subjects did not show obvious signs of depression or anxiety at HA, the lifestyle and cultural changes at HA were different to the one the subjects were used to, which could have affected the subjects’ psychological activity.

In conclusion, this is the first, longitudinal investigation of the ERP at the time of a stay at HA and after subjects return to lowlands. HA hypoxia impaired both visual spatial and non‐spatial tasks. Task induced both the early and late processing stages of ERP components were affected after hypoxic exposure. The affected EEG component was confined in theta power. More complicated spatial information processing seems to be more likely to suffer from hypoxia than non‐spatial information processing. Considering that P3 amplitude was significantly lower in Angle task than Color task and decreased theta power showed a negative correlation with prolonged RT in Angle task but not Color task, inhibitions of electrophysiological components at visual cortex may be better to clarify the deficits of visual spatial discrimination than visual non‐spatial ability.

## DISCLOSURES

No conflicts of interest, financial or otherwise, are declared by the authors.

## AUTHOR CONTRIBUTIONS

Q.Q. and J.Z. conceived and designed the research; P.L., Y.Z., F.Y., X.Z., X.Z., and S.L. performed experiments and analyzed the data; Q.Q. and J.Z. drafted the manuscript; X.L. interpreted the results of experiments; J.Z. approved the final version of manuscript.
